# Adherence to daily, oral TDF/FTC PrEP during periconception among HIV-exposed South African women

**DOI:** 10.3389/frph.2023.1263422

**Published:** 2023-10-04

**Authors:** Kathleen E. Hurwitz, Oluwaseyi O. Isehunwa, Kayla R. Hendrickson, Manjeetha Jaggernath, Yolandie Kriel, Patricia M. Smith, Mxolisi Mathenjwa, Kara Bennett, Christina Psaros, Jared M. Baeten, David R. Bangsberg, Jessica E. Haberer, Jennifer A. Smit, Lynn T. Matthews

**Affiliations:** ^1^ Department of Epidemiology and Statistics, Target RWE, Durham, NC, United States; ^2^ Division of Infectious Diseases, Department of Medicine, University of Alabama at Birmingham, Birmingham, AL, United States; ^3^ MRU (Maternal, Adolescent and Child Health Research Unit), University of the Witwatersrand, Durban, South Africa; ^4^ Department of Epidemiology and Prevention, Centre for the AIDS Programme of Research in South Africa (CAPRISA), Durban, South Africa; ^5^ Department of Psychiatry, Massachusetts General Hospital, Harvard Medical School, Boston, MA, United States; ^6^ Department of Medicine, University of Washington, Seattle, WA, United States; ^7^ Vin University College of Health Sciences, Hanoi, Vietnam; ^8^ Center for Global Health, Massachusetts General Hospital, Boston, MA, United States; ^9^ Department of Medicine, Harvard Medical School, Boston, MA, United States

**Keywords:** HIV prevention, oral pre-exposure prophylaxis (PrEP), adherence, women, periconception, group-based trajectory modeling (GBTM)

## Abstract

**Background:**

Daily, oral tenofovir disoproxil fumarate/emtricitabine (TDF/FTC) as pre-exposure prophylaxis (PrEP) reduces HIV acquisition for African women. Adherence is key to efficacy and patterns of adherence can be highly variable in real-world settings. Using group-based trajectory modeling (GBTM), we sought to identify distinct patterns of periconception PrEP adherence and evaluate potential baseline predictors of such adherence trajectories.

**Methods:**

We conducted a single-arm longitudinal study for women aged 18–35 years living in Durban, South Africa with personal or partner plans for pregnancy with a partner with HIV or of unknown serostatus. Participants were offered safer conception counseling, including daily oral PrEP; women who initiated PrEP were given a bottle with an electronic pillcap that recorded when device opens. Weekly adherence to daily PrEP was modeled using GBTM with a censored normal outcome distribution as a function of weeks since PrEP initiation. The number and functional form of the adherence trajectory groups were primarily selected based on Bayesian information criteria (BIC) and confirmed by mean estimated probabilities of group membership. A multivariable version of the selected model assessed baseline predictors of membership in adherence trajectory groups.

**Results:**

Overall mean (95% CI) adherence to PrEP was 63% (60%, 67%). We identified four groups of women with distinct patterns of adherence: (1) high (i.e., ≥6 doses per week) steady adherence throughout follow-up (22% of PrEP initiators); (2) moderate (i.e., 4–5 doses per week), but steady adherence (31%); (3) initially high, but consistently declining adherence (21%); and (4) initially moderate adherence, followed by a rapid decline and subsequent rebound (26%). In multivariable-adjusted analyses, older age was associated with membership in the high, steady adherence group as compared to the group identified with an adherence trajectory of initially high, then decline, and finally a rebound.

**Conclusions:**

GBTM is useful for exploring potential heterogeneity in longitudinal patterns of medication adherence. Although a large proportion of women in this study achieved high levels of adherence by electronic pillcap initially, far fewer women maintained these levels consistently. Knowledge of different adherence trajectories could be used to develop targeted strategies for optimizing HIV prevention during periconception.

## Introduction

Women of reproductive age constitute the majority of new HIV infections in South Africa (1). It is vital to ensure that women who wish to conceive with a partner with HIV or of unknown HIV-status have access to strategies to lower the risk of HIV acquisition during conception. Daily, oral tenofovir disoproxil fumarate/emtricitabine (TDF/FTC) as pre-exposure prophylaxis (PrEP) is an important individually-controlled method for reducing risk of HIV acquisition during conception and throughout pregnancy (2, 3).

PrEP has high efficacy for HIV protection in African women and is safe to use during pregnancy; however, adherence is key to maintaining protection (4–7). Uptake of and adherence to PrEP in real-world settings is highly variable (8–10). Changes in adherence can be driven by multiple factors including intentional breaks in PrEP use (11) (i.e., no risk of HIV perceived) or gaps in adherence due to challenges overcoming known barriers to use such as medication fatigue, drug side effects, and partner relationship dynamics. This presents an implementation challenge as no gold standard exists for measuring adherence, especially when it changes over time (12, 13). Common approaches to measuring and describing adherence include using prescription refill data or pill bottle openings as a proxy measure of medication consumption and cross-sectional assessments of drug levels to approximate average historical adherence. However, these methods report a single value to summarize medication use and may miss potentially informative trends in the existence and corresponding behavior of sub-groups. Analytic approaches that allow for the identification and subsequent stratification of these sub-groups represent a critical first step to designing effective targeted interventions.

Group-based trajectory modeling (GBTM) is a method first developed in the field of criminology for assessing individual-level differences in criminal career patterns (14–16). The use of GBTM has since been expanded to look at other behavior patterns like medication adherence (12, 17, 18), sexual risk-taking (19), and healthcare expenditures (20). A key advantage of GBTM is that it allows for individual-level heterogeneity in propensity for the outcome without assuming an underlying distribution for that propensity. This both allows for unmeasured variables to impact the outcome and enables the detection of sub-group trajectories within the data (14, 15).

The current analysis uses data collected as part of a single-arm interventional trial in Durban, South Africa to evaluate the use of TDF/FTC as PrEP among women with potential for HIV-exposure and planning for pregnancy (21). A cohort of 330 women aged 18–35 were enrolled and offered safer conception counseling, including daily oral PrEP. Participants were followed for 12-months (or longer if they became pregnant), and those who initiated PrEP were given a pill bottle with an electronic cap that recorded when the device was opened, providing an assessment of day-to-day dosing behavior. Using the electronic pill cap data, we applied GBTM to (a) identify distinct patterns of longitudinal PrEP adherence within trial participants and (b) evaluate potential baseline predictors of any identified adherence trajectory groups.

## Materials and methods

### Study design and population

The Zivikele ngaphambi kokukhulelwa (ZINK) (“Protecting yourself before pregnancy study” in Zulu) was a single-arm longitudinal study conducted in Durban, South Africa. ZINK participants included women aged 18–35 years who tested HIV-negative at enrollment and reported personal or partner plans for pregnancy within the next 12 months with a partner with HIV or of unknown serostatus. All participants were offered safer conception education emphasizing the importance of couples-based HIV counseling and testing, antiretroviral therapy (ART) for partners living with HIV, treatment for sexually transmitted infections (STIs), and safer conception strategies, such as limiting sex without condoms to peak fertility. We also offered daily TDF/FTC, oral PrEP during periconception and pregnancy with adherence support. Recruitment was conducted by field teams reaching out directly to women at local Department of Health (DoH) primary health care clinics within the eThekwini District, gathering spots near the research site, and through word-of-mouth promotion from enrolled participants and others who knew of the study.

The primary aims of the study were to determine PrEP uptake and use during the periconception period and pregnancy. The current manuscript reports on PrEP adherence during periconception (the period from enrollment until study exit or incident pregnancy).

### Study procedures

A complete description of the study procedures including the safer conception education, visit schedules, adherence support, and questionnaires can be found elsewhere (21). Briefly, at the enrollment visit, women were offered a package of safer conception counseling based on South African guidelines (22). Safer conception counseling occurred at baseline and at each quarterly visit thereafter for non-pregnant women.

Participants who chose to use PrEP were offered oral, daily TDF/FTC PrEP. Women were counseled to use PrEP for 7 days (as per WHO 2016 guidance) before engaging in sex without condoms or other backup protection. Women could choose to initiate/discontinue PrEP at any time during the periconception period. At the time of initiation, women were provided with a 30-day supply of PrEP, consistent with South African guidelines at the time. At each quarterly visit thereafter, a 90-day supply was provided.

### Laboratory

Participants completed beta-HCG urine pregnancy testing, individual HIV counseling and testing (HCT), and syndromic screening for STIs at each study visit. Participants who seroconverted were followed to promote linkage to care and conduct genotyping. Participants with a positive pregnancy test were referred to antenatal care and those with STI symptoms were referred to a local clinic for treatment.

Women who chose to initiate PrEP completed blood testing for renal function (creatinine) and for hepatitis B infection consistent with CDC and WHO guidelines at the time. Women with abnormal renal function or active hepatitis B infection were instructed to stop PrEP. Renal function testing was repeated quarterly PrEP could be re-started if serum creatinine and/or eGFR levels returned to normal.

### Questionnaires

Baseline questionnaire was administered to assess constructs within our conceptual framework for periconception risk behavior (e.g., risk perception, reproductive autonomy, HIV stigma) (23, 24) using instruments validated in this setting. Questionnaires were administered via face-to-face interviews with a trained research assistant fluent in English and the dominant local language, isiZulu.

### COVID 19 study adaptations

Due to the COVID-19 pandemic, South Africa was on a nationwide lockdown from March 27, 2020, to May 1, 2020. During that period, telephonic data collection was conducted for study activities to ensure the safety of participants. Only essential clinical visits were allowed and participants were screened for COVID-19 symptoms before their visits. Non-essential in-person clinic visits were allowed for up to 6-months for non-pregnant participants with 6-month PrEP prescriptions.

### Measuring periconception adherence to PrEP

To measure daily pill-taking behavior, women were provided with a pill bottle with an electronic cap [Medication Electronic Monitoring System (MEMS) (AARDEX, Switzerland)] that recorded when the device was opened, providing an objective assessment of day-to-day dosing behavior. Among women who initiated PrEP, defined as collection of at least one month's supply of PrEP within 12 months of enrollment, we assessed adherence to PrEP as the number of days with a time-stamped record of a pill bottle opening (capped at one opening per day) divided by the number of days the participant was in active PrEP follow-up. Women were only monitored for adherence while in active PrEP follow-up, which we define as continued attendance of follow-up visits and acceptance of PrEP refills. For the purposes of defining adherence follow-up, women were censored at the earlier of the following: end of study, positive pregnancy test, HIV seroconversion, lost to follow-up, relocation outside the study area, or withdrawal from study. Women who became pregnant during the study were eligible to continue or start taking PrEP, but for the current analysis we restricted their data to the periconception period due to smaller numbers of pregnant women. A separate analysis will report on PrEP uptake in the pregnant cohort. During our study, PrEP was not widely available in the public health system in South Africa and was contra-indicated among women planning for or with pregnancy. Therefore, we assumed that any participant who missed a refill for any reason was no longer in active PrEP follow-up and could be censored at that time point; however, censored women contributed adherence data up through censorship and were eligible for any adherence pattern.

### Statistical analysis

We used GBTM to identify sub-groups of PrEP initiators who followed distinct adherence trajectories over time. GBTM is an application of discrete mixture modeling that uses maximum likelihood estimation to fit multiple regression models simultaneously (25). More specifically, given the number of potential groups, the procedure fits (a) an intercept-only multinominal logistic regression model for the probability of membership in each group along with (b) separate regression models for the conditional distribution of the longitudinal data (conditional on group) as a smoothed function of time using higher-order polynomials.

For the current study, we modeled weekly adherence to PrEP (range: 0–7 doses per week) using a censored normal distribution as a function of weeks since PrEP initiation. We fit models with 2, 3, 4, and 5 potential adherence groups with up to a third-order polynomial (i.e., cubic term for time). The final model specification—including the number of groups and functional form of each group trajectory—was selected primarily based on the Bayesian information criteria (BIC). That is, models with lower BIC values indicate better fits to the observed data. The output from GBTM not only includes the estimated coefficients for the shape of each trajectory group, but also the estimated probability of group membership per group per participant and the proportion of participants “assigned” to each group. GBTM assigns individuals to groups according to the group with highest estimated probability of membership for that individual. As recommended by Nagin (2005) and Nagin and Odgers (2010), we confirmed the adequacy of the identified model fit by ensuring that the mean estimated probability of group membership for individuals assigned to each group was high (i.e., >0.7) with reasonably narrow confidence intervals (25, 26). To assess the potential for informative censoring, we compared baseline characteristics of women who were lost to follow-up, moved, or withdrew with women who were retained (women who became pregnant or seroconverted were not considered lost, as their censoring was part of the study design).

Lastly, using the selected model specification, we performed multivariable-adjusted analyses to assess predictors of membership in adherence trajectory groups. Candidate predictors were selected *a priori* based on our periconception HIV risk conceptual framework (24) and factors associated with adherence measured by tenofovir concentrations at 3 months (manuscript under review), and included age, education, perceived HIV risk (27), and PrEP optimism (28). All statistical analyses were conducted using SAS software version 9.4 (SAS Institute, Cary, NC, USA) and PROC TRAJ (29).

### Ethics

The protocol was approved by the Human Research Ethics Committee at the University of the Witwatersrand (Pretoria, South Africa) and the Institutional Review Board of Partners Healthcare (Boston, MA, USA) and University of Alabama at Birmingham. The protocol is registered with the South African Health Products Regulatory Agency (SAHPRA), MCC#20170131) and at ClinicalTrials.gov (NCT03194308).

## Results

**Figure 1:**
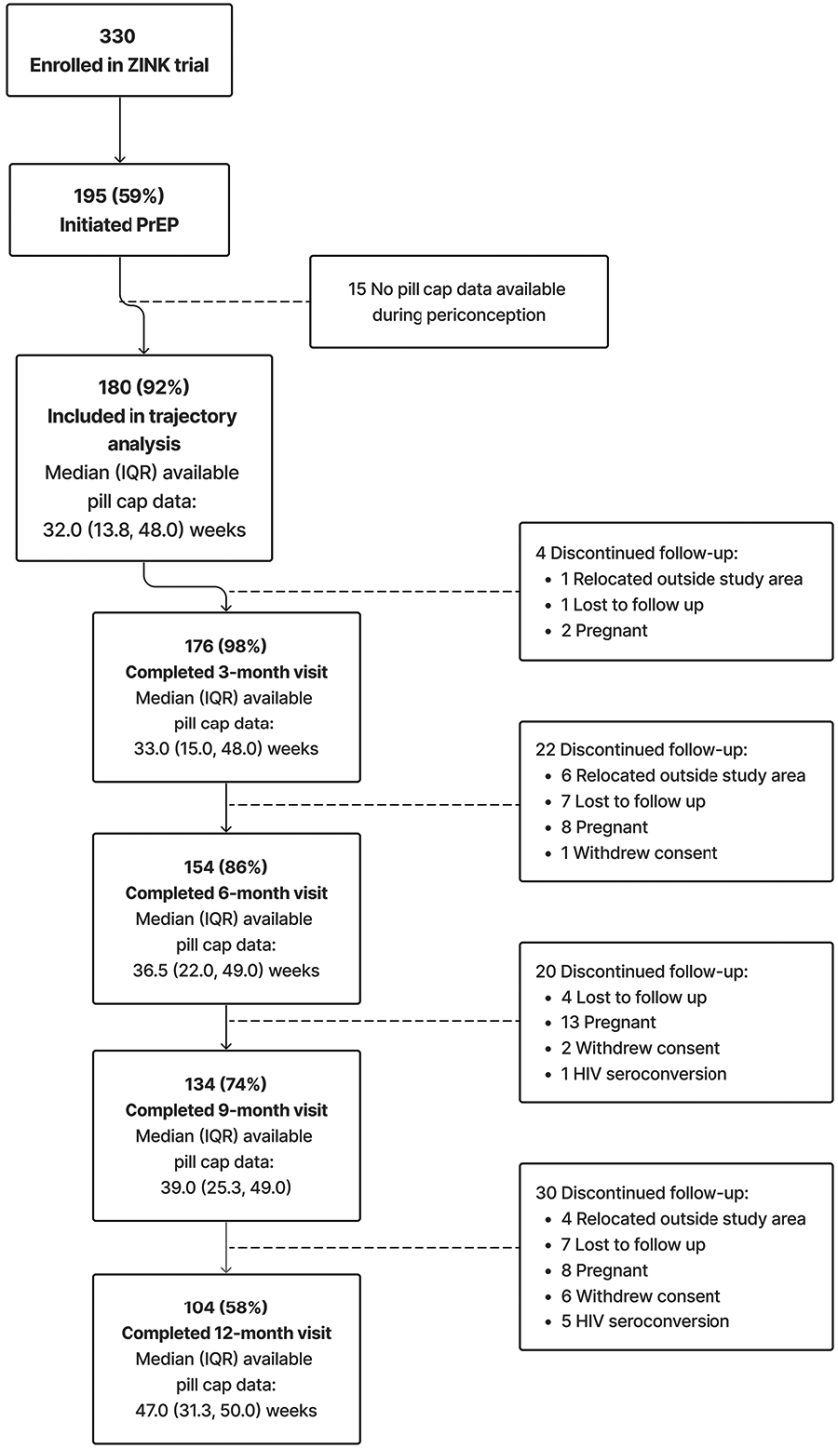
Summary of enrollment, eligibility, and retention of cohort included in trajectory analysis.

Between October 2017 and January 2020, we enrolled 330 women, of whom 195 (59%) initiated PrEP as a primary HIV-prevention method (Figure 1). A total of 180 (92% of PrEP initiators) were included in the current analysis. Fifteen women who initiated PrEP were excluded due to either no available pillcap adherence data (*n* = 13) or no pillcap adherence data prior to first positive pregnancy test result (*n* = 2). Among the 180 PrEP initiators with periconception pillcap adherence data, median (25th, 75th percentile) age was 24.4 (21.7, 27.2) years with 152 (84%) reporting education through or beyond grade 12 and 46 (25%) were employed. Most women (*n* = 110; 61%) had at least one prior pregnancy and 171 (96%) had been in a steady relationship for at least 6 months. Nearly all participants (*n* = 172, 96%) reported not knowing the HIV serostatus of their primary pregnancy partner at enrollment (Table 1).

**Table 1:** Summary of baseline characteristics of *N* = 180 women initiating PrEP and enrolled in a single arm trial of a safer conception intervention for HIV prevention in Durban, South Africa 2017–2020, overall and by adherence trajectory group.

Characteristics (*n* with available data)		Adherence trajectory group^a^			
	Overall *N* = 180	1: High steady adherence *N* = 40 (22%)	2: Moderate but steady adherence *N* = 55 (31%)	3: Consistently declining adherence *N* = 38 (21%)	4: Rapidly declining adherence then rebound *N* = 47 (26%)
Age (years) (*n *= 180)					
Mean	24.8	25.8	25.1	24.8	23.5
Median (25th, 75th percentile)	24.4 (21.7, 27.2)	25.5 (23.1, 29.1)	24.9 (22.8, 26.7)	24.4 (20.9, 27.8)	22.3 (20.7, 25.8)
Education (*n *= 180)					
Grade 7–11	28 (16%)	7 (18%)	7 (13%)	8 (21%)	6 (13%)
Grade 12 or beyond	152 (84%)	33 (83%)	48 (87%)	30 (79%)	41 (87%)
Currently employed (*n *= 180)	46 (25%)	16 (40%)	15 (27%)	7 (18%)	8 (17%)
Income, per month (*n *= 131)^b^					
<$116	48 (36%)	9 (32%)	12 (31%)	11 (41%)	16 (42%)
$116-$232	41 (31%)	11 (39%)	12 (31%)	8 (30%)	10 (26%)
>$232	42 (32%)	8 (28%)	14 (37%)	8 (30%)	12 (31%)
Prior pregnancies (*n *= 180)					
0	70 (39%)	16 (40%)	17 (31%)	17 (45%)	20 (42%)
1	71 (39%)	13 (32%)	23 (42%)	13 (34%)	22 (47%)
2+	39 (22%)	1 (27%)	15 (27%)	8 (21%)	5 (11%)
Sexual partners, past 3 months (*n *= 179)					
1	156 (87%)	39 (97%)	43 (80%)	36 (95%)	38 (81%)
2+	23 (13%)	1 (2%)	11 (20%)	2 (5%)	9 (19%)
HIV serostatus of pregnancy partner (*n *= 179)					
Known to be HIV negative	0 (0%)	0 (0%)	0 (0%)	0 (0%)	0 (0%)
Known to be HIV positive	7 (4%)	6 (15%)	0 (0%)	1 (3%)	0 (0%)
Unknown HIV serostatus	172 (96%)	34 (85%)	54 (100%)	37 (97%)	47 (100%)
Relationship status with pregnancy partner (*n *= 179)					
Ongoing casual partner/one-time encounter	2 (1%)	0 (0%)	1 (2%)	0 (0%)	1 (2%)
Boyfriend/main partner for <6 months	6 (3%)	2 (5%)	0 (0%)	2 (5%)	2 (4%)
Boyfriend/main partner for ≥6 months	164 (92%)	36 (90%)	51 (94%)	35 (92%)	42 (89%)
Spouse or living as married ≥6 months	7 (4%)	2 (5%)	2 (4%)	1 (3%)	2 (4%)
Any alcohol consumption, past year (*n *= 179)	93 (52%)	17 (42%)	33 (61%)	18 (47%)	25 (53%)
Depression score ≥ 1.75 (*n *= 177)^c^	9 (5%)	2 (5%)	5 (9%)	1 (3%)	1 (2%)
Sexual Relationship Power Score (*n *= 156)^d^					
Mean	2.6	2.6	2.6	2.6	2.6
Median (25th, 75th percentile)	2.6 (2.4, 2.8)	2.7 (2.5, 2.9)	2.6 (2.4, 2.8)	2.6 (2.5, 2.8)	2.6 (2.3, 2.8)
Perceived HIV risk (*n *= 170)^e^					
Mean	19.7	20	20	19.7	19.1
Median (25th, 75th percentile)	20 (18, 22)	20 (18, 23)	21 (18, 21)	19.5 (18, 21)	19 (17.5, 21)
PrEP optimism (*n *= 177)^f^					
Mean	5.7	5.9	6	5.3	5.7
Median (25th, 75th percentile)	6.0 (5.0, 7.0)	5.0 (5.0, 7.0)	6.0 (5.0, 7.0)	5.0 (5.0, 6.0)	6.0 (5.0, 6.0)

^a^ *N* (%) unless otherwise noted; column percentages calculated among those with non-missing covariate data.

^b^ Converted to $USD from ZAR.

^c^ Hopkins Symptom Checklist was used to derive depression scores.

^d^ Developed by Pulerwitz et al., the Sexual Relationship Power Score assesses Relationship Control and Decision-Making Dominance between a male and female partner.

^e^ Developed by Napper et al., the HIV-risk score assesses a person's perception of their risk of acquiring HIV based on their sexual and lifestyle habits.

^f^ Adapted from Kalichman SC., the PrEP optimism score assess a person's attitudes about taking PrEP for HIV prevention.

For the current analysis of periconception pillcap adherence, the most common reason for discontinuing follow-up was pregnancy (*n* = 31) followed by loss to follow-up (*n* = 19) and relocation outside the study area (*n* = 11). Six women acquired HIV during follow-up, none of whom had detectable drug concentrations **(**Figure 1). Supplementary Table S1 summarizes the baseline characteristics of women lost to follow up with those who remained under study. Briefly, women lost to follow up were less likely to have prior pregnancies. Other demographics were similar, with women lost to follow up having slightly higher income and education. The total amount of observed PrEP follow-up time ranged from 1 to 52 weeks with most women contributing >32 weeks [overall median (25th, 75th percentile): 32 (13.8, 48.0)] weeks. Among women who completed the 9-month follow-up visit the median (25th, 75th percentile) duration of PrEP follow-up was 39 (25.3, 49.0) weeks (Figure 1). Overall mean (95% CI) adherence to PrEP through 39 weeks was 63% (60%, 67%).

**Figure 2:**
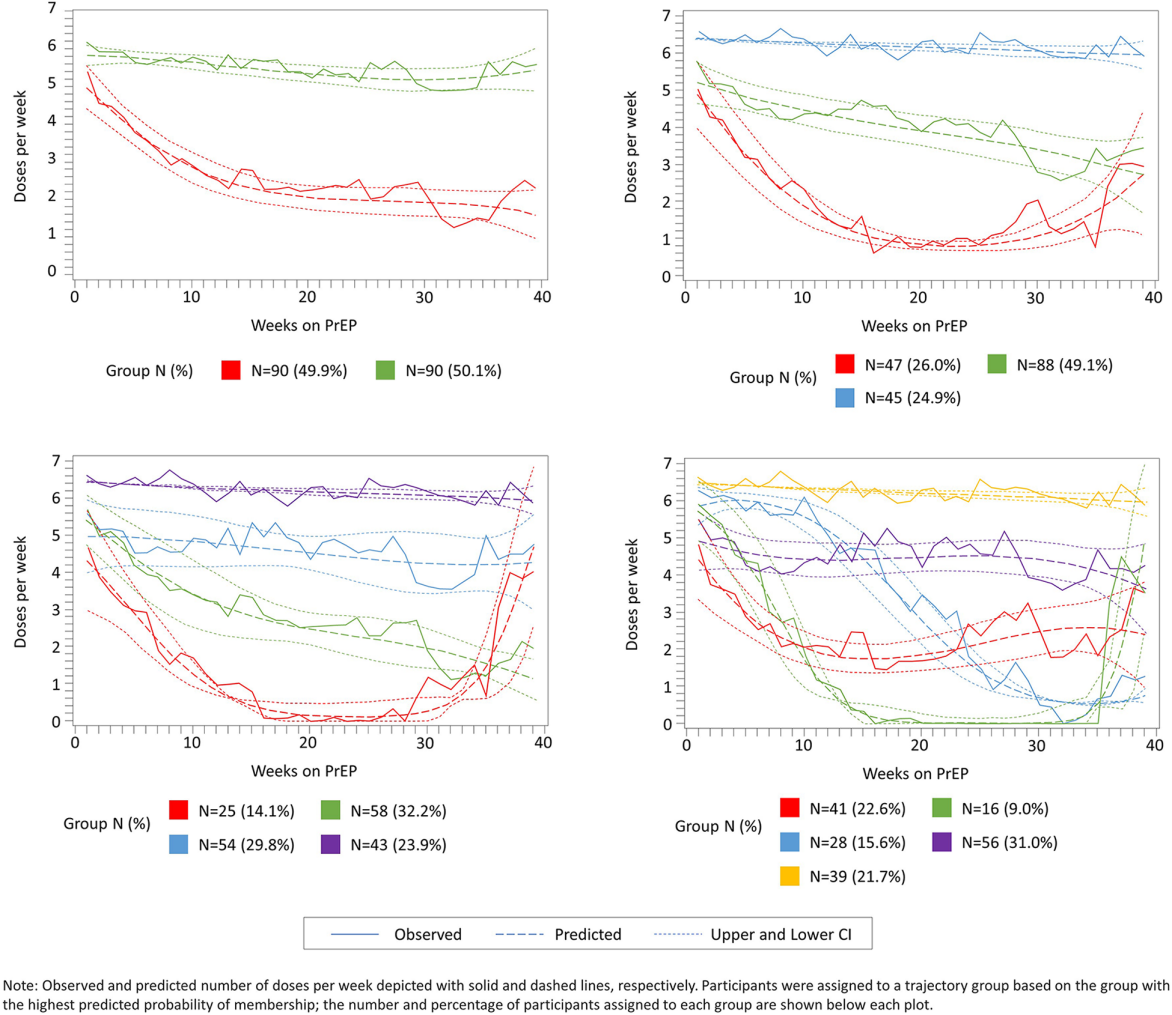
Observed (solid lines) and predicted (dashed lines) weekly adherence to PrEP over 39 months of follow-up assuming 2, 3, 4, and 5 trajectory groups and third-order polynomial terms for time since PrEP initiation.

Figure 2 presents and compares the observed (bold lines) and predicted lines (dashed lines) weekly adherence to PrEP using 2–5 trajectory groups with third-order polynomial terms for time since PrEP initiation. The maximum observed follow-up time for adherence was 52 weeks. However, in analyses using all observed follow-up time, trajectory shapes became highly sensitive to outliers during later time periods. This loss of data was due to multiple reasons, including late initiation of PrEP, administrative censoring of women after pregnancy or seroconversion, and women moving or withdrawing consent. Therefore, we restricted the follow-up period for the current analysis to 39 weeks; corresponding to median adherence follow-up time for women completing the 12-month visit (Figure 1). The 5-group trajectory model was discarded because one group contained <10% of the cohort. A 4-group trajectory model was selected over 2- and 3-group models based on having the lowest BIC value (Supplementary Table S2).

**Figure 3:**
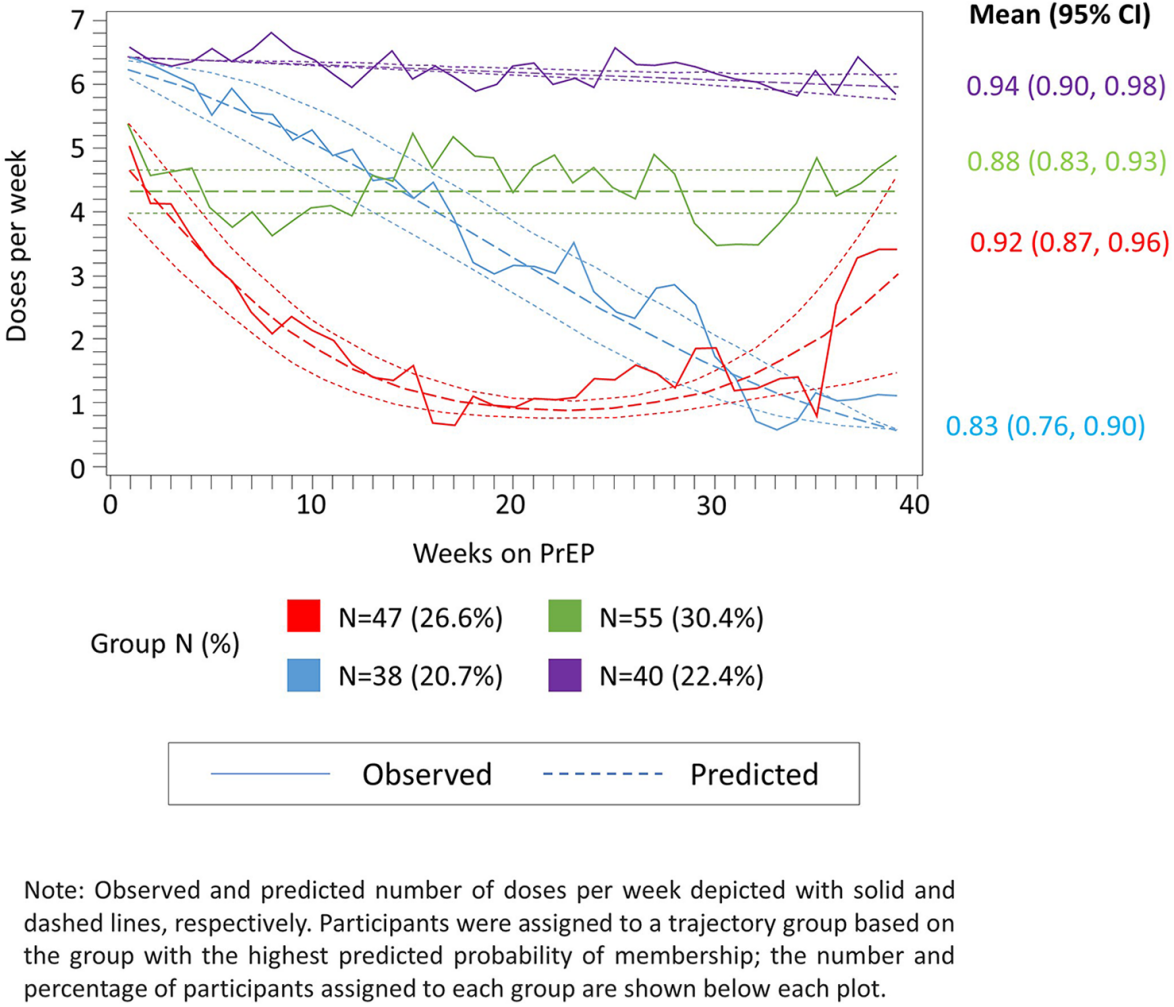
Observed (solid lines) and predicted (dashed lines) weekly adherence to PrEP over 39 months of follow-up assuming four trajectory groups and up to second-order polynomial terms for time since PrEP initiation.

From the final 4-group model specification, we identified groups of women with (1) high (i.e., ≥6 doses per week) steady adherence over time (22% of PrEP initiators); (2) moderate (i.e., 4–5 doses per week), but steady adherence (31%); (3) initially high, but consistently declining adherence (21%); and (4) initially moderate adherence, followed by a rapid decline and subsequent rebound (26%) (Figure 3). The mean predicted probability of group membership for women assigned to each trajectory group was consistently high (all >0.7), indicating adequate model fit. Additionally, the width of the 95% CIs for the mean predicted probability of group membership was reasonably narrow with the widest CI observed for trajectory group 3 (95% CI: 0.76, 0.90 for an absolute width of 0.14). The lower bound for all CIs was above the suggested threshold of 0.7, also confirming the adequacy of model fit (Figure 3).

Table 1 summarizes the baseline characteristics of the adherence cohort overall and according to assigned trajectory group. Women with the high or moderate, but steady adherence over time (i.e., trajectory groups 1 and 2, respectively) were older, more likely to be currently employed, and report higher levels of optimism for PrEP at enrollment as compared to women with consistently or rapidly declining levels of adherence (trajectory groups 3 and 4, respectively). Among women assigned to trajectory groups 2 and 4, approximately 20% reported multiple sexual partners during the past three months, compared with less than 6% reporting the same in groups 1 and 3. Perceived HIV risk and sexual relationship power scores were similar across all four trajectory groups (Table 1).

Table 2 presents and compares a subset of baseline individual predictors (i.e., age, education, perceived HIV risk, and PrEP optimism) of group membership modeled using a multivariable-adjusted version of GBTM. In multivariable-adjusted analyses, age was inversely associated with membership in trajectory group (4) (i.e., initially moderately high adherence, followed by a rapid decline and subsequent rebound trajectory group). More specifically, one additional year of age was associated with a 19% decreased probability of membership in trajectory group (4) as compared with trajectory group (1) (i.e., high steady adherence) (*P *= 0.01). The other two adherence trajectory groups also had negative point estimates for age (i.e., decreased probability of membership as compared to trajectory group (1) (i.e., high steady adherence); however, neither were statistically significant. None of the other baseline covariates we examined were associated with membership in an adherence trajectory group.

**Table 2:** Summary of multivariable-adjusted mean change in probability of membership in an adherence trajectory group among *N* = 180 women initiating PrEP and enrolled in a single-arm trial of a safer conception intervention for HIV prevention in Durban, South Africa 2017–2020.

	Estimate (95% CI) and *P*-value					
	2: Moderate but steady adherence		3: Consistently declining adherence		4: Rapidly declining adherence then rebound	
	(vs. 1: High steady adherence)					
Baseline characteristic						
Age (years)	−0.04 (−0.16, 0.08)	0.46	−0.07 (−0.23, 0.09)	0.34	−0.19 (−0.33, −0.05)	0.01
Education	0.69 (−0.58, 1.96)	0.29	0.11 (−1.36, 1.58)	0.88	0.02 (−1.41, 1.45)	0.98
Perceived HIV-risk	0.002 (−0.16, 0.16)	0.98	−0.03 (−0.23, 0.17)	0.76	−0.14 (−0.32, 0.04)	0.13
PrEP optimism	0.01 (−0.26, 0.28)	0.97	−0.23 (−0.60, 0.14)	0.23	−0.08 (−0.23, 0.39)	0.62

## Discussion

Among women living in an HIV endemic area and planning pregnancy with either a partner with HIV or of unknown serostatus, most chose PrEP as a safer conception strategy. These data indicate high demand for and acceptability of periconception PrEP in South Africa. The overall adherence summary suggests mean adherence by pillcap was 63% (corresponding to approximately 4.4 doses per week on average); our analysis of these data exposes that this mean includes women with excellent adherence (≥6 pills per week), moderate (4–5 pills per week), and two groups comprised of women who did not sustain adherence longitudinally, with one group having some rebound around 30 weeks of follow-up. Importantly, over half (52%) of those taking PrEP consistently took 4–6 doses per week over the follow-up period. Without GBTM, we miss important heterogeneity in how women planning for pregnancy use PrEP. Understanding and being able to identify these distinct adherence patterns may inform future efforts to tailor support for women accessing PrEP.

A few studies described patterns of PrEP adherence among women of reproductive age using GBTM with different results (12, 13, 30). An open-label demonstration project conducted in Kenya and Uganda used GBTM to identify four patterns of PrEP adherence (via daily electronic monitoring) among 233 women in HIV-serodifferent partnerships: high steady adherence, moderate steady, late declining, and early declining adherence (13). Approximately 55% of women were consistently and highly adherent. This is consistent with prior studies indicating that many women in mutually-disclosed HIV-serodifferent partnerships are successful in overcoming challenges in maintaining high adherence to PrEP (6, 31) A separate PrEP demonstration project conducted in Kenya—The Monitoring PrEP among Young Adult women (MPYA) study—used GBTM to identify three PrEP adherence patterns among 348 women aged 18–24 years: steady high adherence, moderate but declining, and low and declining (12). In contrast to the current study, only 5% of women exhibited steady, high adherence. The higher proportion of moderate to high PrEP adherence patterns in our study may result from motivations to achieve pregnancy while avoiding HIV and/or the reproductive-goals focused adherence support counseling, Healthy Families-PrEP, that was offered (21, 32).

Over half of our participants accessing PrEP consistently took 4–6 doses per week with about one-fifth taking at least 6 doses per week. Women in the other 2 groups were distinguished within 12 weeks of monitoring. We believe this early distinction in PrEP use could be a useful target for future interventions. The lone sociodemographic characteristic significantly associated with trajectory group was age: older women were more likely to display steady high levels of adherence to PrEP. This result is consistent with previously published findings (9). However, a more nuanced understanding of why young women experience worsening PrEP adherence over time will help to further optimize existing HIV prevention programs. In addition, over a quarter of women who had declining PrEP use experienced a rebound around 30 weeks of follow-up. Women participated in quarterly adherence support; it is possible that the 6-month session boosted use. It is also possible that life circumstances changed for this group (perhaps evolving decisions re. pregnancy plans). Future qualitative work to understand PrEP use by trajectory could be used to better understand factors informing how women choose to use PrEP and thus optimize support strategies offered. PrEP adherence support groups or interventions could be personalized especially for early or late PrEP decliners. Additionally, implementation studies that prioritize identifying PrEP adherence patterns early on, as well as developing optimal strategies for women with early declining adherence patterns would be desirable.

A key strength of this study is the combination of electronic monitoring of PrEP adherence via the MEMS caps and the GBTM analysis method. Using a measure of daily adherence allows for sufficiently granular data to make trajectory analysis possible. This contrasts with other measures of adherence, such as weekly or monthly prescription refill data, or drug concentration data collected at intervals. Although MEMS caps are not without measurement error, they have been demonstrated as more reliable than self-reported adherence, and the data correspond well with biomarkers of tenofovir intake (33). Another strength is that while our study population allowed for the inclusion of women who knew their partner was living with HIV, most women (96%) were unaware of the HIV status of their partner, making the findings more generalizable to women who live or interact in high HIV prevalence communities without concrete knowledge of their partner's HIV status. A limitation of this study is that our small sample size may have inhibited us from detecting meaningful demographic differences between the trajectory groups (12, 13). In addition, this analysis was limited to periconception PrEP use due to smaller numbers of pregnant women accessing PrEP (which will be addressed in a separate analysis).

In conclusion, women of reproductive age in HIV endemic regions remain susceptible to acquiring HIV. PrEP is an effective biomedical HIV prevention intervention when used consistently, however adherence remains a challenge. GBTM is a useful method for assessing how sub-groups of a population split into different patterns of longitudinal medication adherence. We found evidence of four different patterns of PrEP adherence behavior among a prospective cohort of women with potential for HIV exposure planning pregnancy, and age was associated with being in the rapid decline trajectory group. Nearly half of the women did not sustain steady adherence over the follow-up period. These findings indicate that younger women planning for pregnancy may be at risk of not adhering to PrEP over time and may benefit from novel strategies that address their unique needs and adherence barriers. Further research should explore the possibility of developing risk scores based on early adherence patterns to screen for women who may be at risk for declining adherence. Finally, given that current guidelines in South Africa are permissive of continuing PrEP during pregnancy, future implementation studies stake should into consideration different PrEP adherence trajectories among African women throughout their reproductive cycle to inform models of support.

## Data Availability

The datasets presented in this article are not readily available because primary analyses are ongoing. Once final analyses are complete, data will be shared to the Harvard Dataverse. In the interim, requests to access the datasets should be directed to the corresponding author. Requests to access the datasets should be directed to LM: lynnmatthews@uabmc.edu.
