# Supplementary material for: Adherence to daily, oral TDF/FTC PrEP during periconception among HIV-exposed South African women
Source: Front Reprod Health. 2023 Oct 4;5:1263422. doi: 10.3389/frph.2023.1263422 (PMC10582627; doi:10.3389/frph.2023.1263422)
Supplement: Supplementary file 1 [file Table1.docx]

| **Supplemental Table 1.** Comparison of baseline characteristics between women lost to follow-up (LFTU) and women who completed analysis follow-up | | | |
| --- | --- | --- | --- |
|  | **Overall (N=180)** | **Completed study analysis follow-up (N=141)** | **LTFU  (N=39)** |
| **Age (years)** (*n*=180) |  |  |  |
| Mean | 24.8 | 24.9 | 24.3 |
| Median (25^th^, 75^th^ percentile) | 24.3  (21.7, 27.1) | 24.4  (21.9, 27.2) | 23.5  (21.1, 26.4) |
| **Education** (*n*=180) |  |  |  |
| Grade 7 - 11 | 28 (15.6%) | 25 (17.7%) | 3 (7.7%) |
| Grade 12 or beyond | 152 (84.4%) | 116 (82.3%) | 36 (92.3%) |
| **Currently employed** (*n*=180) |  |  |  |
| No | 134 (74.4%) | 103 (73.0%) | 31 (79.5%) |
| Yes | 46 (25.6%) | 38 (27.0%) | 8 (20.5%) |
| **Income, per month** (*n*=131) |  |  |  |
| < $116 | 48 (36.6%) | 41 (39.0%) | 7 (26.9%) |
| $116 - $232 | 41 (31.3%) | 28 (26.7%) | 13 (50.0%) |
| > $232 | 42 (32.1%) | 36 (34.3%) | 6 (23.1%) |
| **Prior pregnancies** (*n*=180) |  |  |  |
| 0 | 70 (38.9%) | 49 (34.8%) | 21 (53.8%) |
| 1 | 71 (39.4%) | 59 (41.8%) | 12 (30.8%) |
| 2+ | 39 (21.7%) | 33 (23.4%) | 6 (15.4%) |
| **Sexual partners, past 3 months** (*n*=179) |  |  |  |
| 0 | 0 (0%) | 0 (0%) | 0 (0%) |
| 1 | 156 (87.2%) | 124 (88.6%) | 32 (82.1%) |
| 2+ | 23 (12.8%) | 16 (11.4%) | 7 (17.9%) |
| **HIV serostatus of pregnancy partner** (*n*=179) |  |  |  |
| Known to be HIV negative | 0 (0%) | 0 (0%) | 0 (0%) |
| Known to be HIV positive | 7 (3.9%) | 6 (4.3%) | 1 (2.6%) |
| Unknown HIV serostatus | 172 (96.1%) | 134 (95.7%) | 38 (97.4%) |
| **Relationship status with pregnancy partner** (*n*=179) |  |  |  |
| Ongoing casual partner/one-time encounter | 2 (1.1%) | 1 (0.7%) | 1 (2.6%) |
| Boyfriend/main partner for <6 months | 6 (3.4%) | 3 (2.1%) | 3 (7.7%) |
| Boyfriend/main partner for >=6 months | 164 (91.6%) | 132 (94.3%) | 32 (82.1%) |
| Spouse or living as married >=6 months | 7 (3.9%) | 4 (2.9%) | 3 (7.7%) |
| **Any alcohol consumption, past year** (*n*=179) |  |  |  |
| Never | 86 (48.0%) | 66 (47.1%) | 20 (51.3%) |
| Any Amount | 93 (52.0%) | 74 (52.9%) | 19 (48.7%) |
| **Depression score >= 1.75** (*n*=177) |  |  |  |
| <= 1.75 | 168 (94.9%) | 133 (95.7%) | 35 (92.1%) |
| > 1.75 | 9 (5.1%) | 6 (4.3%) | 3 (7.9%) |
| **Sexual relationship power** (*n*=156) |  |  |  |
| Mean | 2.60 | 2.59 | 2.60 |
| Median [Q1, Q3] | 2.61  (2.39, 2.76) | 2.61  (2.38, 2.76) | 2.56  (2.42, 2.77) |
| **Perceived HIV risk** (*n*=170) |  |  |  |
| Mean | 19.7 | 19.7 | 19.8 |
| Median (25^th^, 75^th^ percentile) | 20.0  (18.0, 22.0) | 20.0  (18.0, 21.5) | 20.0  (17.0, 22.0) |
| **PrEP optimism** (*n*=177) |  |  |  |
| Mean | 5.72 | 5.75 | 5.62 |
| Median (25^th^, 75^th^ percentile) | 6.00  (5.00, 7.00) | 6.00  (5.00, 7.00) | 5.00  (5.00, 6.00) |
