# Supplementary material for: Adherence to daily, oral TDF/FTC PrEP during periconception among HIV-exposed South African women
Source: Front Reprod Health. 2023 Oct 4;5:1263422. doi: 10.3389/frph.2023.1263422 (PMC10582627; doi:10.3389/frph.2023.1263422)
Supplement: Supplementary file 2 [file Table2.docx]

| Supplementary Table 2:  Summary of Bayesian Information Criteria and Group Membership according to the assumed number and functional form of the trajectory groups. | | | | | | | | |
| --- | --- | --- | --- | --- | --- | --- | --- | --- |
|  | | | | **Percentage of Women in Each Group** | | | | |
| Number of groups | Trajectory shapes | BIC^2^  N=4838 | BIC^3^  N=180 | 1 | 2 | 3 | 4 | 5 |
| 2 | 2 0 | -9134.3 | -9124.4 | 49.1 | 50.9 | - | - | - |
| 2 | 2 1 | -9125.1 | -9113.6 | 49.7 | 50.3 | - | - | - |
| 2 | 2 2 | -9127.0 | -9113.8 | 49.8 | 50.2 | - | - | - |
| 3 | 2 0 0 | -8947.5 | -8934.3 | 37.7 | 36 | 26.2 | - | - |
| 3 | 2 0 1 | -8946.1 | -8931.3 | 37.9 | 36.1 | 26 | - | - |
| 3 | 2 0 2 | -8950.2 | -8933.8 | 37.9 | 36.1 | 26 | - | - |
| 3 | 2 1 0 | -8913.1 | -8898.3 | 26.1 | 48.2 | 25.8 | - | - |
| 3 | 2 1 1 | -8912.5 | -8896.1 | 26.4 | 48.9 | 24.8 | - | - |
| 3 | 2 1 2 | -8916.7 | -8898.6 | 26.4 | 48.9 | 24.7 | - | - |
| 3 | 2 2 0 | -8917.2 | -8900.7 | 26.0 | 48.4 | 25.6 | - | - |
| 3 | 2 2 1 | -8916.6 | -8898.5 | 26.2 | 49.1 | 24.7 | - | - |
| 3 | 2 2 2 | -8920.8 | 8901.0 | 26.2 | 49.1 | 24.7 | - | - |
| 4 | 2 0 0 0 | -8873.9 | -8854.1 | 27.6 | 16.4 | 31.5 | 24.4 | - |
| 4 | 2 0 0 1 | -8869.8 | -8851.6 | 27.6 | 16.4 | 31.5 | 24.5 | - |
| 4 | 2 0 0 2 | -8873.9 | -8854.1 | 27.6 | 16.4 | 31.5 | 24.4 | - |
| 4 | 2 0 1 0 | -8784.9 | -8766.8 | 26.6 | 30.4 | 20.3 | 22.7 | - |
| 4 | 2 0 1 1 | -8783.2 | -8763.5 | 26.6 | 30.4 | 20.7 | 22.4 | - |
| 4 | 2 0 1 2 | -8787.5 | -8766.1 | 26.6 | 30.4 | 20.7 | 22.4 | - |
| 4 | 2 0 2 0 | -8788.5 | -8768.8 | 26.4 | 30.3 | 20.6 | 22.7 | - |
| 4 | 2 0 2 1 | -8786.9 | -8765.5 | 26.4 | 30.2 | 21 | 22.4 | - |
| 4 | 2 0 2 2 | -8791.1 | -8768.1 | 26.4 | 30.2 | 21 | 22.4 | - |
| 4 | 2 1 0 0 | -9013.7 | -8995.6 | 0.0 | 38.7 | 34.9 | 26.4 | - |
| 4 | 2 1 0 1 | -9012.5 | -8992.8 | 0.0 | 39.0 | 35.2 | 25.8 | - |
| 4 | 2 1 0 2 | -8787.5 | -8766.1 | 26.6 | 20.7 | 30.4 | 22.4 | - |
| 4 | 2 1 1 0 | -8997.5 | -8977.8 | 0.0 | 29.8 | 45.5 | 24.7 | - |
| 4 | 2 1 1 1 | -8996.3 | -8974.9 | 0.0 | 30.0 | 46.1 | 24.0 | - |
| 4 | 2 1 1 2 | -8791.2 | -8968.1 | 26.4 | 18.9 | 32.4 | 22.3 | - |
| 4 | 2 1 2 0 | -8793.4 | -8772.0 | 21.9 | 30.6 | 24.1 | 23.4 | - |
| 4 | 2 1 2 1 | -8790.7 | -8767.7 | 23.4 | 35.3 | 18.8 | 22.4 | - |
| 4 | 2 1 2 2 | -8794.9 | -8770.2 | 23.4 | 35.3 | 18.8 | 22.4 | - |
| 4 | 2 2 1 0 | -8793.4 | -8772.0 | 21.9 | 24.1 | 30.6 | 23.4 | - |
| 4 | 2 2 1 1 | -8790.5 | -8767.5 | 26.1 | 18.5 | 33.1 | 22.3 | - |
| 4 | 2 2 1 2 | -8794.7 | -8770.1 | 26.1 | 18.5 | 33.1 | 22.3 | - |
| 4 | 2 2 2 0 | -8797.6 | -8774.6 | 21.9 | 24.1 | 30.6 | 23.4 | - |
| 4 | 2 2 2 1 | -8794.7 | -8770.0 | 26.1 | 18.5 | 33.1 | 22.3 | - |
| 4 | 2 2 2 2 | -8799.0 | -8772.6 | 26.1 | 18.5 | 33.1 | 22.3 | - |
| 5 | 2 2 1 0 0 | -8714.0 | -8689.3 | 10.1 | 21.4 | 18.9 | 27.1 | 22.5 |
| 5 | 2 2 1 0 1 | -8712.1 | -8686.8 | 10.1 | 21.4 | 19.2 | 27.2 | 22.0 |
| 5 | 2 2 1 0 2 | -8758.7 | -8730.7 | 26.1 | 5.5 | 18.1 | 28.3 | 22.0 |
| 5 | 2 2 1 1 0 | -8717.9 | -8691.6 | 10.1 | 21.6 | 18.5 | 27.2 | 22.5 |
| 5 | 2 2 1 1 1 | -8716.0 | -8688.1 | 10.1 | 21.6 | 18.8 | 27.4 | 22.0 |
| 5 | 2 2 1 1 2 | -8767.3 | -8737.7 | 23.8 | 6.5 | 19.0 | 28.5 | 22.1 |
| 5 | 2 2 2 0 0 | -8718.1 | -8691.8 | 21.5 | 10.1 | 18.9 | 27.0 | 22.5 |
| 5 | 2 2 2 0 1 | -8763.2 | -8735.2 | 23.9 | 6.5 | 19.1 | 28.4 | 22.1 |
| 5 | 2 2 2 0 2 | -8767.4 | -8737.8 | 23.9 | 6.5 | 19.1 | 28.4 | 22.1 |
| 5 | 2 2 2 1 0 | -8722.1 | -8694.1 | 21.7 | 10.1 | 18.6 | 27.1 | 22.5 |
| 5 | 2 2 2 1 1 | -8767.4 | -8737.7 | 23.9 | 6.5 | 19.0 | 28.5 | 22.1 |
| 5 | 2 2 2 1 2 | -8771.6 | -8740.3 | 23.9 | 6.5 | 19.0 | 28.5 | 22.1 |
| 5 | 2 2 2 2 0 | -8726.3 | -8696.7 | 21.7 | 10.1 | 18.6 | 27.1 | 22.4 |
| 5 | 2 2 2 2 1 | -8724.4 | -8693.2 | 21.7 | 10.1 | 18.9 | 27.2 | 22.0 |
| 5 | 2 2 2 2 2 | -8775.8 | -8742.9 | 23.9 | 6.6 | 19.0 | 28.5 | 22.1 |
